# *Christensenella minuta*, a new candidate next-generation probiotic: current evidence and future trajectories

**DOI:** 10.3389/fmicb.2023.1241259

**Published:** 2024-01-11

**Authors:** Olga Ignatyeva, Darya Tolyneva, Aleksandr Kovalyov, Lorena Matkava, Mikhail Terekhov, Daria Kashtanova, Anzhelika Zagainova, Mikhail Ivanov, Vladimir Yudin, Valentin Makarov, Anton Keskinov, Sergey Kraevoy, Sergey Yudin

**Affiliations:** Centre for Strategic Planning and Management of Biomedical Health Risks, Federal Biomedical Agency, Moscow, Russia

**Keywords:** *Christensenellaceae*, *Christensenella*, probiotics, metabolic disorders, inflammatory bowel diseases

## Abstract

**Background:**

As the field of probiotic research continues to expand, new beneficial strains are being discovered. The *Christensenellaceae* family and its newly described member, *Christensenella minuta*, have been shown to offer great health benefits. We aimed to extensively review the existing literature on these microorganisms to highlight the advantages of their use as probiotics and address some of the most challenging aspects of their commercial production and potential solutions.

**Methods:**

We applied a simple search algorithm using the key words “*Christensenellaceae*” and “*Christensenella minuta*” to find all articles reporting the biotherapeutic effects of these microorganisms. Only articles reporting evidence-based results were reviewed.

**Results:**

The review showed that *Christensenella minuta* has demonstrated numerous beneficial properties and a wider range of uses than previously thought. Moreover, it has been shown to be oxygen-tolerant, which is an immense advantage in the manufacturing and production of *Christensenella minuta*-based biotherapeutics. The results suggest that *Christensenellaceae* and *Christensenella munita* specifically can play a crucial role in maintaining a healthy gut microbiome. Furthermore, *Christensenellaceae* have been associated with weight management. Preliminary studies suggest that this probiotic strain could have a positive impact on metabolic disorders like diabetes and obesity, as well as inflammatory bowel disease.

**Conclusion:**

*Christensenellaceae* and *Christensenella munita* specifically offer immense health benefits and could be used in the management and therapy of a wide range of health conditions. In addition to the impressive biotherapeutic effect, *Christensenella munita* is oxygen-tolerant, which facilitates commercial production and storage.

## 1 Introduction

We are home to trillions of microorganisms. They live virtually on and in every part of our bodies, affecting our metabolism, immune system, and many vital physiological functions. One could even argue that this coexistence and, hence, coevolution have shaped our species. These highly complex, self-regulating microbial communities form the microbiome, which is often referred to as one of the body’s organs. It has long been considered a promising therapeutic target that can be regulated with probiotics, or “good microorganisms”.

Probiotic foods such as fermented dairy products became a staple long before people were aware of the benefits they offered. The first deliberate efforts to “harness the power” of the microbiome came soon after the successful isolation of several bacterial species from human feces. This newly acquired ability to isolate, culture, and produce beneficial bacterial strains ushered in the era of probiotic drugs. Since then, they have proven effective in treating many infectious and non-communicable diseases.

Most common probiotic drugs contain various strains of lyophilized lactobacilli and/or bifidobacteria; less frequently, *Streptococcus*, *Bacillus*, and others. Since the first successful cultivation of fecal microorganisms, these bacteria have long been considered to be the most important representatives of the human gut microbiome. More recently, the advent of culture-independent molecular techniques, such as polymerase chain reaction (PCR) and especially DNA sequencing, has allowed for a much closer examination of the commensal bacteria. These scientific advances enabled the detection of hundreds of new microorganisms that could not be detected using conventional techniques. Many of these microorganisms were eventually successfully cultured and studied in more detail. More importantly, some of these new representatives of the human microbiome, such as *Akkermansia*, *Faecalibacterium*, *Christensenella*, *Anaerobutyricum*, *Bacteroides*, *Roseburia*, etc., have proven crucial to human health and well-being. This could represent a breakthrough in the history of probiotic medicine and pave the way for next-generation probiotics (NGPs).

In this review, we present an overview of the *Christensenellaceae* family and focus on *Christensenella minuta* (*C. minuta*) as an NGP candidate that could offer tremendous health benefits.

## 2 *Christensenella minuta*: a newly identified member of the human gut microbiome

*C. minuta* (DSM 22607) was first isolated from the feces of a healthy Japanese donor by Morotomi et al. (2012). It was designated as the first member of the new *Christensenellaceae* family in the *Clostridiales* order in the *Firmicutes* phylum. The genus name honors Professor Henrik Christensen, an outstanding Danish microbiologist. The species name reflects the small size of the bacterial cells and their colonies (Morotomi et al., 2012).

**Figure 1:**
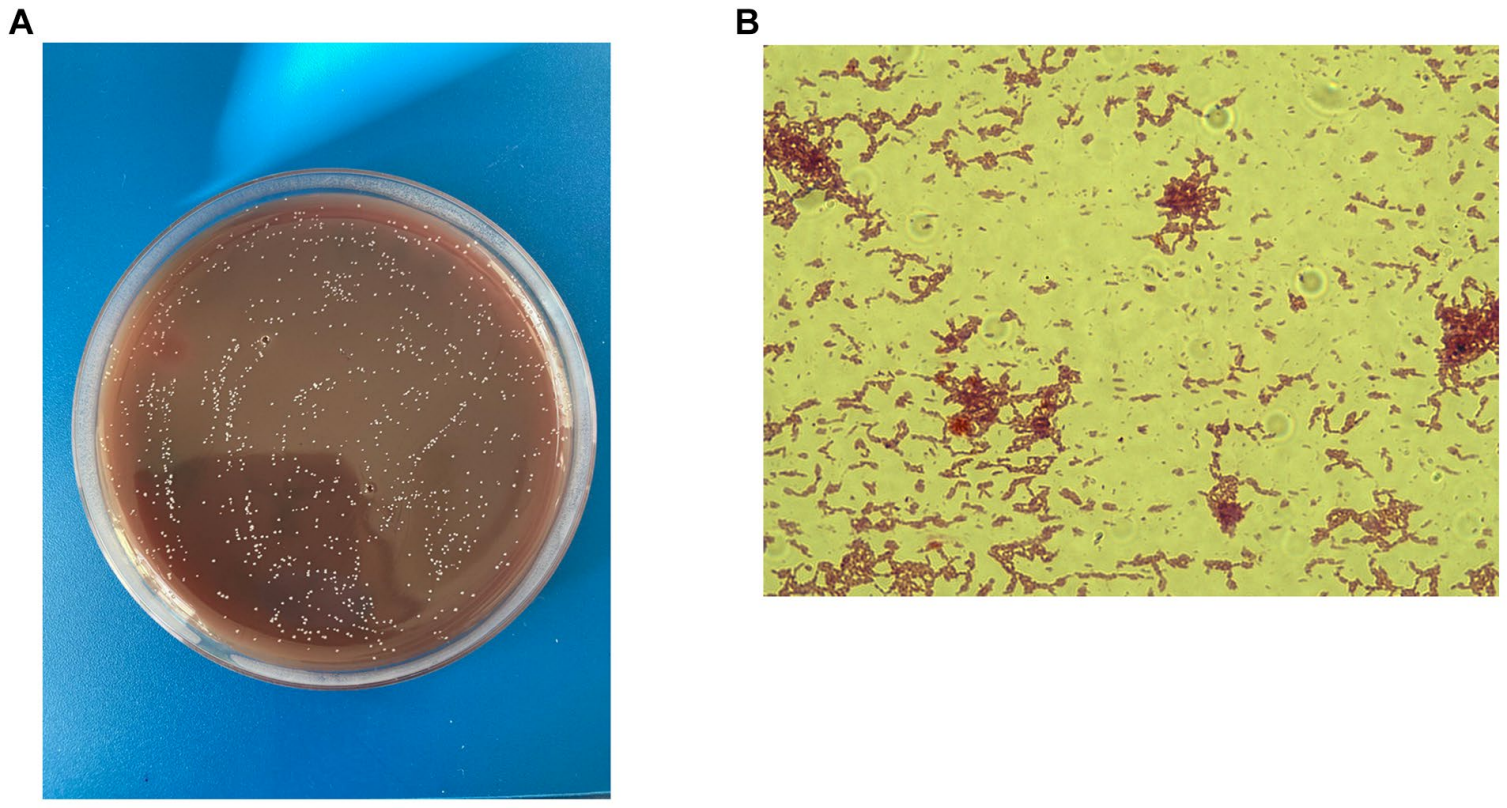
*C. minuta* VKM B-3687D isolated at the Centre for Strategic Planning and Management of Biomedical Health Risks: **(A)** Gram staining; x 100 and **(B)** colonies grown on the Schaedler agar.

*C. minuta* is a small (ranging in size from 0.5 mm to 1.9 mm), gram-negative, non-sporulating, and non-motile bacterium (Figure 1A) that forms circular, almost colorless colonies (Figure 1B). It was originally described as strictly anaerobic; however, later studies demonstrated that it can tolerate oxygen for several hours (Kropp et al., 2021). Contrary to popular belief, exposure to atmospheric air does not instantly kill the bacterium but rather reduces its viability. *C. minuta* grows most rapidly at 37–40° and pH 7.5 (Morotomi et al., 2012). It is not particularly fastidious and can grow on various media, such as Gifu Anaerobic Medium (GAM Broth), Wilkins–Chalgren Anaerobe Agar, and Schaedler Anaerobe Agar (Morotomi et al., 2012).

*C. minuta* can ferment many sugars, including glucose, salicin, xylose, arabinose, rhamnose, and mannose (Morotomi et al., 2012). The main fermentation products include acetic and butyric acids. Additionally, it has been demonstrated to convert organic substrates via fermentation to create large volumes of H_2_ (Ruaud et al., 2020). *C. minuta* does not produce catalase, oxidase, urease, or indole, and does not reduce nitrates (Morotomi et al., 2012). Recently, DSM 22607, one of *C. minuta* strains, has been shown to produce a novel bile salt hydrolase (BSH), which could expand the biotherapeutic range of this microorganism (Dejean et al., 2021).

## 3 The global presence of *Christensenellaceae*

Metagenomic studies have shown that members of the family *Christensenellaceae* are found on all continents (Escobar et al., 2014; Morton et al., 2015; Oki et al., 2016; Barrett et al., 2018; Brooks et al., 2018). They live in the microbiome of a wide variety of animals, from cockroaches (Richards et al., 2017) and lizards (Baldo et al., 2018) to birds (Crisol-Martinez et al., 2017) and mammals (Kamke et al., 2016; McKenzie et al., 2017), including humans. These bacteria are primarily found in the gastrointestinal tract but have also been found in the respiratory tract (Huang et al., 2010; Gangneux et al., 2020) and genitourinary tract in primates (Adapen et al., 2022).

Another potential therapeutic advantage of *Christensenellaceae* is their high heritability, as demonstrated in a large-scale UK study on the gut microbiome of twins (Goodrich et al., 2014). This family has also been described as the hub of a co-occurrence network, i.e., the one with the largest number of connections with other highly heritable taxa. This finding has several implications. First, members of the family *Christensenellaceae* are “hardwired” into the host’s metabolism. Second, they are likely to affect the abundance of other taxa and promote the growth of beneficial commensals such as *Methanobacteriaceae*. Therefore, this low-abundance family, accounting for just about 0.01% of the human microbiome (Goodrich et al., 2014), may hypothetically be a crucial microbiome “builder” and health promoter.

Numerous studies have shown that *Christensenellaceae* are abundant in the healthy microbiome, suggesting that their abundance could be a reliable health marker and a promising biotherapeutic target.

## 4 *Christensenellaceae* in health and disease

**Figure 2:**
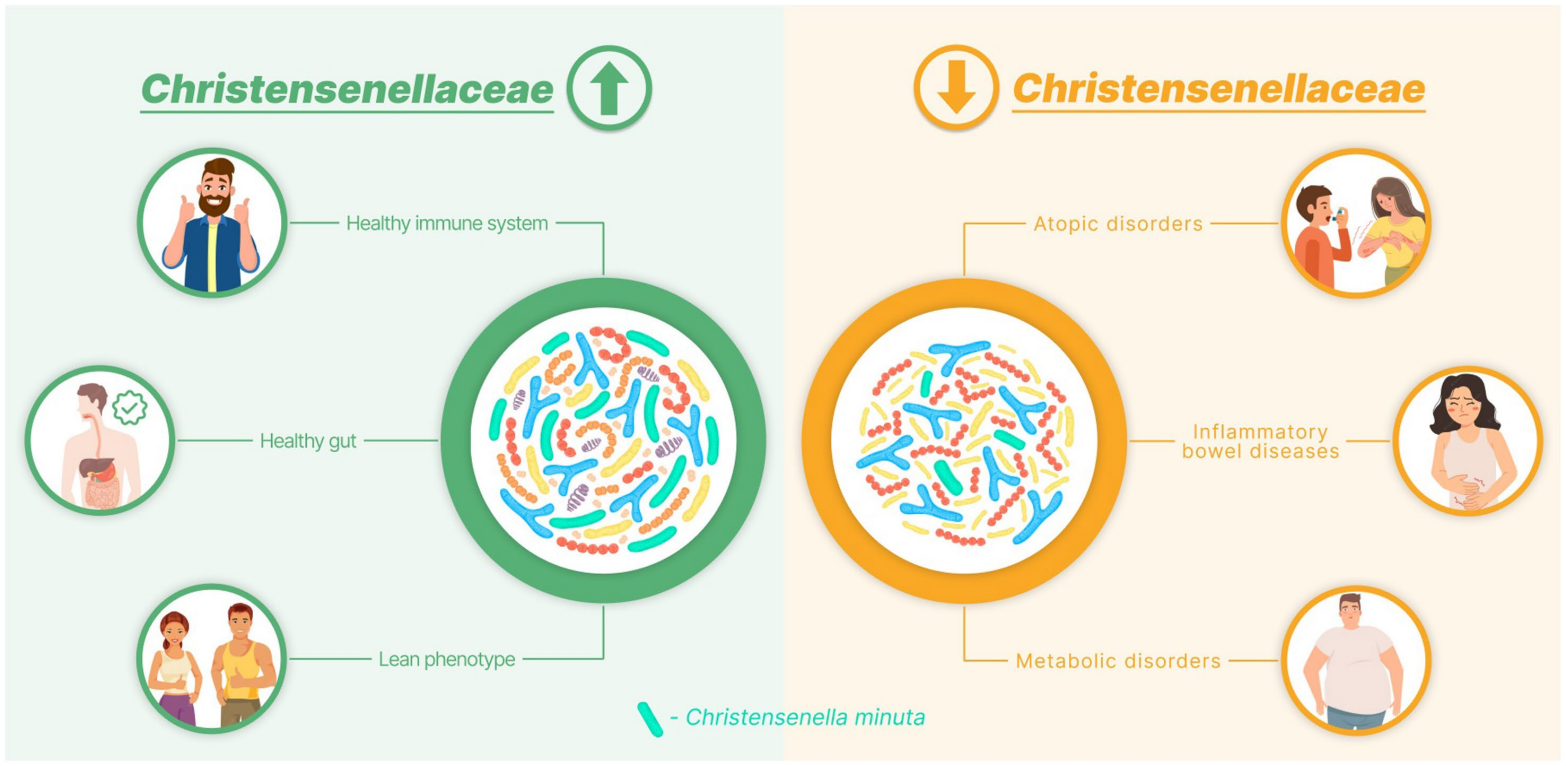
Diagram demonstrating the association between the *Christensenellaceae* abundance and human health.

The *Christensenellaceae* family was isolated and included in the reference databases in 2012. Since then, the benefits offered by its members have been recognized worldwide. This family has been repeatedly described as the “microbial satellite” of health and leanness (Goodrich et al., 2014). It has also been linked to healthy aging (Biagi et al., 2016). Within just 10 years, ample evidence has been accumulated, indicating that *Christensenellaceae* are significantly depleted in many diseases (Figure 2).

The *Christensenellaceae* family has been highlighted in several studies of human metabolism and its markers. Metabolic syndrome and obesity have been commonly reported as disorders associated with *Christensenellaceae* deficiency in both adults (Lim et al., 2017; He et al., 2018; Peters et al., 2018) and children (Alcazar et al., 2022). Multiple pieces of evidence indicate that the abundance of this family correlates with numerous metabolic markers. Sowah et al. detected a negative correlation between the abundance of *Christensenellaceae* in the gut microbiome and several metabolic indicators, such as body mass index (BMI), visceral adipose tissue (VAT), liver fat, insulin resistance (HOMA-IR), and serum levels of triglycerides (Sowah et al., 2022). Other studies have shown that *Christensenellaceae* negatively correlate with total cholesterol, low-density lipoproteins, and apolipoprotein B (He et al., 2018; Lopez-Contreras et al., 2018). The family was underrepresented in patients with atherogenic dyslipidemia (Lopez-Montoya et al., 2022). Not surprisingly, depletion of *Christensenellaceae* has been observed in pathologies normally associated with metabolic syndrome, such as hypertension (Gomez-Arango et al., 2016; Calderon-Perez et al., 2020), prediabetes (Org et al., 2017; Jayanama et al., 2022), and diabetic retinopathy (Liu et al., 2022).

The second most common group of disorders associated with levels of *Christensenellaceae* are gastrointestinal pathologies, primarily inflammatory bowel diseases (IBDs). Numerous studies have documented the depletion of *Christensenellaceae* in patients with Crohn’s disease (CD) and ulcerative colitis (UC) (Pascal et al., 2017; Imhann et al., 2018; Kennedy et al., 2018; Zakrzewski et al., 2019; Teofani et al., 2022). Interestingly, a significant decrease in the abundance of *Christensenellaceae* was observed in patients with quiescent CD immediately prior to flares (Braun et al., 2019), possibly indicating their role in disease progression. A lower abundance of *Christensenellaceae* was also observed in patients with diarrhea (Brunkwall et al., 2021).

*Christensenellaceae* have also been linked to bronchial asthma and atopic diseases. Hu et al. found that children whose fecal microbiome was rich in *Christensenellaceae* were less likely to develop eczema and sensitization to inhalant allergens (Hu et al., 2021). Remarkably, the abundance of *Christensenellaceae* in the household environment may play an important role in bronchial asthma. Gangneux et al. analyzed the metagenomic profiles of dust collected from the houses of healthy children and children and adults with asthma. They found that *Christensenellaceae* was significantly overrepresented in the dust from “healthy” houses, whereas it was depleted in the dust from “asthmatic” houses (Gangneux et al., 2020). This is hardly direct evidence of the impact of *Christensenellaceae* in dust on the likelihood of developing asthma. However, this peculiar finding may reflect differences in the microbial profiles of healthy people and those with bronchial asthma.

Kidney stones (Stanford et al., 2020), affective disorders (Vinberg et al., 2019; Coello et al., 2021), thyroid cancer (Lu et al., 2022), mucous membrane pemphigoid (Low et al., 2022), polycystic ovary syndrome (Dong et al., 2021), and recurrent aphthous stomatitis (Wang et al., 2022) have also been linked to a lower abundance of *Christensenellaceae*.

However, a higher abundance of *Christensenellaceae* is not generally associated with a healthy phenotype. There is evidence, though scarce, of a possible link between this family and various pathologies. In a study by Xu et al., a higher abundance of *Christensenellaceae* increased the risk of death in critically ill patients with neurological disorders admitted to an intensive care unit (Xu et al., 2019). This taxon was significantly enriched in patients with Parkinson’s disease (Shen et al., 2021) and particularly in those with poor clinical profiles (Barichella et al., 2019). An increased abundance of *Christensenellaceae* has also been observed in people with Alzheimer’s disease (Verhaar et al., 2021; Kaiyrlykyzy et al., 2022), multiple sclerosis (Galluzzo et al., 2021), and anorexia nervosa (Prochazkova et al., 2021).

Despite the numerous documented beneficial properties of this microorganism, there is evidence to suggest that *C. minuta* and neurocognitive health could be negatively correlated. As an active producer of short-chain fatty acids, *C. minuta* would be expected to have a positive effect on the gut-brain axis and, by extension, neurocognitive status. However, this may not always be the case. Waters and Ley (2019) also noted this surprising correlation and hypothesized it could be linked to delayed gastrointestinal transit time and constipation commonly seen in patients with Parkinson’s disease and multiple sclerosis (Wiesel et al., 2001; Pedrosa Carrasco et al., 2018). Notably, increased abundance of *Christensenellaceae* was positively correlated with prolonged transit time (Oki et al., 2016; Roager et al., 2016), providing insight into the relationship between this microorganism and neurocogntive status. Therefore, the observed correlation between elevated *Christensenellaceae* levels and neurodegenerative conditions may be indirect, with an alternative pathway directly linking the two.

Information on the prevalence of *C. minuta* in health and disease is summarized in Table 1.

**Table 1:** Summary of the studies reporting the association between the *Christensenellaceae* family and a healthy phenotype or disorder.

Reference	Year	Disorder	Population	Specimens	Abundance of *Christensenellaceae*
Lim et al. (2017)	2017	Metabolic syndrome	Adults 53 monozygotic twin pairs (*n* = 306) and 37 dizygotic twin pairs (*n* = 74), and their parents and siblings (*n* = 275)	Feces	↓ in people with metabolic syndrome
He et al. (2018)	2018	Metabolic syndrome	Adults ~7,000 subjects	Feces	↓ in people with metabolic syndrome
Peters et al. (2018)	2018	Obesity	Adults 599 subjects	Feces	↓ in obese patients
Alcazar et al. (2022)	2022	Obesity	Children 191 children with obesity, including 106 metabolically healthy and 85 metabolically unhealthy	Feces	↓ in metabolically unhealthy children and those with one or more altered cardiovascular risk factors
Sowah et al. (2022)	2022	Overweight and obesity	Adults 147 subjects followed up for 50 weeks within an interventional study	Feces	Negative correlation with BMI, VAT, liver fat, HOMA-IR, and blood levels of triglycerides, and cholesterol
Lopez-Contreras et al. (2018)	2018	Overweight and obesity	Children 67 normal-weight and 71 obese children	Feces	↓ in overweight and obese children
Calderon-Perez et al. (2020)	2020	Hypertension	Adults 29 non-treated hypertensive and 32 normotensive subjects	Feces	↓ in hypertensive subjects
Gomez-Arango et al. (2016)	2016	Hypertension in early pregnancy	Adults 205 women at 16 weeks gestation from the SPRING study	Feces	Negatively correlation with systolic blood pressure
Org et al. (2017)	2017	Metabolic syndrome, pre-diabetes	Adults 531 individuals from the METSIM cohort	Feces	Negatively correlation with serum triglyceride level ↓ in prediabetic subjects
Jayanama et al. (2022)	2022	Prediabetes (HIV infected)	Adults 20 prediabetic subjects vs. 20 normoglycemic subjects	Feces	↓ in prediabetic subjects
Liu et al. (2022)	2022	Diabetic retinopathy	Adults 14,584 patients with diabetic retinopathy vs. 202,082 healthy controls	Feces	↓ in patients with diabetic retinopathy
Imhann et al. (2018)	2018	CD	Adults 188 CD patients vs. 582 healthy controls	Feces	↓ in CD patients
Teofani et al. (2022)	2022	CD, UC	Adults 52 CD patients and 58 UC patients vs. 42 healthy controls	Feces	↓ in both CD and UC patients
Kennedy et al. (2018)	2018	CD	Adults 37 CD patients vs. 54 healthy controls (including 24 household controls without CD)	Feces	↓ in CD patients
Pascal et al. (2017)	2017	CD, UC	Adults 34 CD patients and 33 UC patients vs. 111 healthy controls	Feces	↓ in CD patients, but not in UC patients
Zakrzewski et al. (2019)	2019	CD	Adults 15 CD patients vs. 58 healthy controls	Feces	↓ in the wt group as compared to the group carrying protective variant of the *IL23R* gene
Braun et al. (2019)	2019	CD	Adults 45 CD patients with 217 samples collected over 2 years vs. 22 healthy controls	Feces	↓ in preflare patients
Brunkwall et al. (2021)	2021	Self-reported bowel symptoms	Adults 1988 participants of the Malmö Offspring Study	Feces	↓ in patients with diarrhea
Hu et al. (2021)	2021	Atopic diseases	Children 1,440 subjects	Feces	Negative correlation with risks of eczema, inhalant allergic sensitization, and physician-diagnosed inhalant allergy
Gangneux et al. (2020)	2020	Bronchial asthma	15 dwellings of asthma patients vs. 15 dwellings of healthy controls	Dust	↓ in dwellings of asthma patients
Stanford et al. (2020)	2020	Kidney disease/stones (systematic review)	Adults 892 subjects with kidney disease or kidney stones and 1,400 healthy controls	Feces	↓ in patients with kidney stones
Coello et al. (2021)	2021	Affective disorders	Adults 176 subjects with affective disorders, 70 unaffected relatives, and 101 healthy controls	Feces	↓ in patients with affective disorders
Vinberg et al. (2019)	2019	Affective disorders	Adults 128 monozygotic twins	Feces	↓ in twins with affective disorders
Lu et al. (2022)	2022	Thyroid cancer	Adults 50 patients with thyroid cancer vs. 58 healthy controls	Feces	↓ in patients with thyroid cancer
Dong et al. (2021)	2021	Polycystic ovary syndrome	Adults 45 patients with polycystic ovary syndrome and 37 healthy women	Feces	↓ in patients with polycystic ovary syndrome
Wang et al. (2022)	2022	Recurrent aphthous stomatitis (RAS)	Adults 31 non-smoking RAS patients, 19 smoking RAS patients, and 28 non-smoking healthy controls	Saliva	↓ in RAS patients
Xu et al. (2019)	2019	Patients of the neurological intensive care unit	Adults 98 neurocritically ill patients vs. 84 healthy controls	Feces	Positively associated with an increased risk of death
Shen et al. (2021)	2021	PD	Adults meta-analysis of 15 case-control studies involving 959 PD patients and 744 healthy controls	Feces	↑ in PD patients
Barichella et al. (2019)	2019	PD	Adults 193 idiopathic PD patients stratified by disease duration, 22 patients with progressive supranuclear palsy, 22 patients with multiple system atrophy, 113 and healthy controls	Feces	↑ in PD patients
Kaiyrlykyzy et al. (2022)	2022	AD	Adults 41 AD patients vs. 43 healthy controls	Feces	↑ in AD patients positive correlation with worse nonmotor symptoms
Verhaar et al. (2021)	2022	AD	Adults 33 with patients with AD dementia, 21 patients with mild cognitive impairment, and 116 patients with subjective cognitive decline	Feces	Associated with higher odds of amyloid positivity
Galluzzo et al. (2021)	2021	MS	Adults 15 MS patients vs. 15 healthy controls	Feces	↑ in MS patients
Prochazkova et al. (2021)	2021	Anorexia nervosa	Adults 59 patients with anorexia nervosa vs. 67 healthy controls	Feces	↑ in patients with anorexia nervosa

CD, Crohn’s disease; UC, ulcerative colitis; PD, Parkinson’s disease; AD, Alzheimer’s disease; MS, multiple sclerosis; BMI, body mass index; VAT, visceral adipose tissue; HOMA-IR, homeostasis model assessment-estimated insulin resistance.

## 5 Current evidence of the probiotic activity of *Christensenella minuta*

The above metagenomic studies demonstrated that *Christensenellaceae* could be a source of a highly potent probiotic drug that would benefit many patient groups, particularly those with metabolic disorders and inflammatory gastrointestinal pathologies. This inspired new research into the properties and therapeutic capacity of *C. minuta*.

All mechanisms underlying the probiotic activity of *C. minuta* have yet to be fully understood; however, there have been many positive developments. Kropp et al. tested *C. minuta* DSM 22607 in a series of *in vitro* and *in vivo* experiments, and their results were extremely promising. First, both the bacterium and its supernatant demonstrated a strong anti-inflammatory potential provided by their ability to limit IL-8 production in HT-29 cells. The supernatant also inhibited the NF-kB signaling pathway, whereas the bacterium did not. Second, *C. minuta* also showed the ability to protect the intestinal barrier in TNF-α-compromised Caco-2 cells, as demonstrated by transepithelial electrical resistance (TEER) (Kropp et al., 2021).

These results were confirmed in murine models of dinitrobenzene sulfonic acid (DNBS)- and trinitrobenzene sulfonic acid (TNBS)-induced colitis. In both experiments, *C. minuta* displayed distinctive anti-inflammatory properties and protected the colonic tissues as effectively as 5-aminosalicylic acid (5-ASA). The bacterium reduced both macroscopic and microscopic chemical damage, decreased immune cell infiltration (ICI) in the colon, limited oxidative stress, and lowered the secretion of pro-inflammatory cytokines and the expression of lipocalin-2 (Kropp et al., 2021).

The experiments also shed some light on the metabolic phenotype of *C. minuta*, particularly its ability to produce large amounts of acetate and moderate amounts of butyrate, which supports the findings of Morotomi et al. (Kropp et al., 2021). Importantly, *C. minuta* has been reported to produce both short-chain fatty acids (SCFAs), while most microorganisms can only produce butyrate or acetate.

Relizani et al. isolated and tested thirty-two new *C. minuta* strains from nine donors to identify the best candidate probiotic drug. They analyzed the anti-inflammatory and protective properties of these strains in a series of experiments and selected five main candidates. All five candidates prevented NF-kB pathway activation after TNF-α stimulation and induced IL-10 production in *in vitro* cell models. In animal models, two of the five strains significantly improved the inflammatory lesions caused by TNBC and had pronounced local anti-inflammatory effects. In addition, the authors demonstrated the ability of the *C. minuta* strains to stimulate IL-10 production by human-derived PBMC in an *in vitro* model (Relizani et al., 2022).

Mazier et al. conducted a thorough investigation of the anti-obesity potential of *C. minuta* DSM22607 (Mazier et al., 2021). They found that daily administration of 2 × 10^9^ colony-forming units (CFU) of *C. minuta* prevented weight gain and hyperglycemia in mice fed a high-fat diet (HFD) but did not affect their food intake. Surprisingly, there was no statistically significant difference in weight gain between the animals consuming the probiotic strain and the animals feeding on normal chow; however, the HFD-fed mice receiving the vehicle demonstrated a significant and rapid weight gain. This strongly suggests that *C. minuta* limited fat accumulation by altering metabolism rather than by affecting feeding behavior. These findings correlated with the observations made on serum metabolic markers, namely a decrease in leptin and resistin levels in HFD mice. *C. minuta* may have disrupted hepatic lipogenesis, as demonstrated by a reduced expression of the *Gck* gene encoding glucokinase. In addition, the probiotic strain had a strong protective effect on gut permeability by upregulating the *Ocln* and *Zo1* genes, which encode major tight-junction proteins. This may also have contributed to *C. minuta*’s anti-obesity effect by limiting systemic inflammation caused by leaky gut.

In the same study (Mazier et al., 2021), the authors also attempted to assess the effects of a *C. minuta* biotherapeutic on gut microbiome composition in a murine model and in a SHIME^®^ model inoculated with human feces from obese individuals. In mice daily gavaged with *C. minuta* DSM22607, its abundance in feces reached 20% on average, which resulted in a lower alpha-diversity compared with the controls. To avoid bias, the authors excluded the *Christensenellaceae* family from the analysis and found that the microbial profile in the HFD-DSM22607 mice fell somewhere between those of the mice fed with normal chow and the mice fed with an HFD vehicle, with no significant difference in beta-diversity. Meanwhile, the microbial profiles of these two animal groups differed significantly. The authors concluded that *C. minuta* tended to limit HFD-related shifts in the gut microbiome. Some valuable results were observed in the SHIME^®^ model. *C. minuta* failed to engraft in the microbial ecosystem of this *in vitro* gut model after the treatment; it, however, improved the microbial diversity in the “distal colon” compartment and significantly enriched both compartments with SCFAs, which was sustained even during the washout period. Finally, *C. minuta* increased the levels of litocholic acid and unconjugated bile salt acid, indicating the production of a bile salt hydrolase.

Pan et al. have further corroborated the evidence for the beneficial effects of *C. minuta* and its critical role in metabolic processes. The authors used two strains of the genus *Christensenella* (*C. minuta* DSM 22607 and *C. timonesis* DSM 102800) to treat streptozotocin (STZ)-induced type 2 diabetes mellitus in mice. Numerous metabolic indicators were improved by both strains. The gavaged probiotic bacteria lowered blood glucose levels, limited oxidative stress, promoted the restoration of damaged pancreatic islet and liver cells, and suppressed the expression of several pro-inflammatory cytokines and TLR4 in the liver and colon. Importantly, the treatment with *C. minuta* and *C. timonesis* also upregulated Zonula occludens-1 and Claudin-1 in the colon, thereby strengthening the intestinal barrier. This finding was supported by the decreased serum LPS levels in the test subjects. Both strains also had a profound effect on metabolism by stimulating the expression of proglucagon, increasing serum levels of glucagon-like peptide-1 (GLP-1), and limiting hepatic gluconeogenesis. Finally, both *Christensenella* strains altered the composition of the gut microbiome by increasing the abundance of many beneficial microorganisms, such as *Bifidobacterium* and *Phascolarctobacterium*. Overall, *C. minuta* and *C. timonesis* improved metabolic processes in T2DM mice and mitigated inflammatory responses (Pan et al., 2022).

We can only hypothesize about the exact mechanisms underlying the beneficial effects of the *Christensenella* species. However, all of the above studies have laid a solid foundation for further research.

## 6 Possible mechanisms underlying the probiotic effects of *Christensenella minuta*

Based on the above results, we hypothesized about the mechanisms that could underlie the effects of the *Christensenella* family and its metabolites, including the ability to control inflammation, improve metabolism, and modulate the entire microbiome. Here, we will attempt to summarize what is known about *Christensenella* and outline some possible research trajectories and their implications.

### 6.1 Short-chain fatty acids

Short-chain fatty acids (SCFAs) are primarily derived from the fermentation of indigestible carbohydrates by gut microbes, including *C. minuta*. This species produces acetate and butyrate (Kropp et al., 2021), valuable metabolites acting as postbiotics, which likely contribute to the therapeutic effects of *C. minuta*. Numerous studies have demonstrated the beneficial effects of SCFAs both locally in the intestine and at the systemic level.

Acetate and butyrate can serve as fuel for adenosine triphosphate (ATP) in intestinal epithelial cells and support homeostasis. Moreover, both SCFAs synthesized by *C. minuta* are presumably involved in maintaining the intestinal epithelial barrier. For example, acetate and butyrate can activate the nucleotide-binding oligomerization domain 3 (NLRP3) inflammasome by binding to G-protein-coupled receptors GPR43 and GPR109A. This increases IL-18 release, facilitating the repair of epithelial cells (Macia et al., 2015). Butyrate stabilizes hypoxia-inducible factor (HIF) (Kelly and Colgan, 2016), a key molecule for barrier protection and tissue regeneration, upregulates tight junction proteins (Peng et al., 2009; Wang et al., 2012; Miao et al., 2016), and increases mucin production by goblet cells (Gaudier et al., 2004), resulting in a stronger intestinal barrier. Interestingly, butyrate can also have the opposite effect. Kaiko et al. found that butyrate can inhibit the proliferation of intestinal stem cells through the forkhead box O3 (Foxo3) (Kaiko et al., 2016). On the one hand, this impedes epithelial regeneration and wound healing. On the other hand, it can be a control mechanism for excessive cell division and tumor growth prevention. Therefore, as a key producer of such an important metabolite, *C. minuta* could offer additional benefits.

The beneficial effects of SCFAs are not limited to the intestinal barrier. Many authors have reported their immunomodulating potential. For instance, both acetate and butyrate showed their ability to inhibit LPS-induced release of tumor necrosis factor-α (TNF-α) and interferon-γ (IFN-γ) in human leucocytes (Cox et al., 2009). In the study by Park et al., butyrate inhibited IFN-γ-induced production of nitric oxide synthase (NOS), TNF-α, and IL-6 in murine macrophages and increased IL-10 release, which indicates a high anti-inflammatory potency of this SCFA (Park et al., 2016). Butyrate could also suppress the LPS-induced production of chemokines, namely CCL3, CCL4, CCL5, CXCL9, CXCL10, and CXCL11, thus affecting leukocyte trafficking and limiting the production of IL-6 and IL-12p40 (Nastasi et al., 2015). It has also been demonstrated to promote regulatory T-cell production and differentiation, which is critical for immune regulation and homeostasis (Arpaia et al., 2013).

The profiles of acetate and butyrate would not be complete without mentioning their contribution to the gut-brain axis and their metabolic effects. These SCFAs, produced by the gut microbiome, interact with enteroendocrine cells in the colonic mucosa and induce the release of glucagon-like peptide-1 (GLP-1) and peptide YY (PYY) (Freeland and Wolever, 2010; Christiansen et al., 2018), known as anorexic hormones. These hormones enter the systemic circulation and exert their effects on many organs and tissues, most importantly – in the stomach and pancreas. Together, GLP-1 and PYY prevent rapid gastric emptying, inhibit acid secretion and motility, and slow gastrointestinal transit, which collectively results in poor appetite and reduced food intake. GLP-1 also stimulates insulin secretion and prevents β-cell exhaustion in the pancreas (Rondas et al., 2013).

### 6.2 Bile salt hydrolase activity

Bile salt hydrolases (BSHs) are a group of bacterial enzymes involved in the biotransformation of bile acids (BAs). BAs are produced in the liver by cholesterol oxidation, then stored in the gallbladder, and finally excreted into the duodenum after food intake. Before reaching the intestine, BAs are conjugated to either taurine or glycine, making them less hydrophobic and less toxic. After fulfilling their function, most BAs are reabsorbed and migrate to the liver, where they can be reused. However, about 5% of BAs are deconjugated in the gut by enzymes produced by BSH-competent commensal microorganisms, such as *C. minuta*, and eventually excreted in the feces. Dejean et al. showed that two strains of *C. minuta* (DSM33407 and DSM) carried an active BSH that can hydrolyze both taurine- and glycine-conjugated BAs, with DSM being more active (Dejean et al., 2021).

The relationship between the gut microbiome and BAs could be essential to both. After their deconjugation, BAs become significantly more toxic and can affect or even shape bacterial communities in the gut. These communities, in turn, can cause changes in BA metabolism and extend the effects of BAs well beyond the intestine. Gadaleta et al. hypothesized that BSH-mediated deconjugation of BAs can, to some extent, disrupt BA recycling. As a result, BAs are reabsorbed and transported to the liver at a much slower rate, while their *de novo* synthesis increases, consuming cholesterol and lowering its levels in blood serum (Gadaleta et al., 2022). The exact mechanisms underlying the effect of microbiome-derived BSH have yet to be fully understood; however, there is increasing evidence that BAs and the gut microbiome interact very closely and are both affected.

### 6.3 Lipopolysaccharides

The mystery of *C. minuta*’s anti-inflammatory properties is likely to be explained, at least in part, by its unusual lipopolysaccharides (LPSs). Yang et al. conducted a detailed assessment of LPSs in *C. minuta* and obtained interesting results. They compared the genetics of LPS biosynthesis in *E. coli* and *C. minuta*; *C. minuta* lacked some genes encoding key virulence-related LPSs, suggesting that its LPSs might be less toxic. Furthermore, *C. minuta* presumably has a shorter O-antigen repeat chain, which is important for LPS recognition by the immune system. Sodium dodecyl sulfate-polyacrylamide gel electrophoresis (SDS-PAGE) of LPSs in *E. coli* and *C. minuta* confirmed the differences in their structures (Yang et al., 2018). The authors also examined the effect of LPSs in *E. coli* and *C. minuta* on murine macrophages. LPSs from both bacteria demonstrated the ability to stimulate proliferation and phagocytosis. However, LPSs of *E. coli* were effective even at much lower concentrations. Then, the authors measured the expression of key proteins involved in the NF-κB pathway after the macrophages were exposed to LPSs and found that *C. minuta* LPSs were a relatively weak trigger of this important inflammation pathway. Furthermore, LPS-induced production of cytokines, nitric oxide, and reactive oxygen species was substantially lower in the macrophages stimulated by *C. minuta* LPS than in those stimulated by *E. coli* LPS (Yang et al., 2018).

Therefore, *C. minuta* LPSs, being distinctively different from those of more common gram-negative bacteria, have lower ability to elicit innate immune responses by interacting with toll-like receptors (TLRs). These findings may explain some of the effects observed in the experiments by Kropp et al. and Relizani et al., but they do not clarify the mechanism underlying the production of anti-inflammatory cytokines. Overall, the study by Yang et al. is an important step towards solving the mystery of *C. minuta*.

### 6.4 Bacterial associations

The beneficial effects of *C. minuta* and the *Christensenellaceae* family are likely due to their specific ability to interact with numerous other bacterial communities in the gut. Besides its direct effects, *C. minuta* can also affect the host indirectly by promoting or restricting the growth of certain taxa. The co-occurrence network constructed by Li et al. revealed that the *Christensenellaceae* family was positively correlated with many taxa, including *Oscillospira*, *Ruminococcus*, *Coprococcus*, *Prevotella*, *Akkermansia*, *Roseburia*, etc. In contrast, several genera such as *Klebsiella*, *Streptococcus*, *Fusobacterium*, *Magamonas*, and *Blautia* were inversely correlated with *Christensenellaceae*. Furthermore, there was a significant association between a higher abundance of *Christensenellaceae* and increased microbial richness and diversity (Li et al., 2020). Notably, several of the co-occurring taxa have been proposed as new-generation probiotics (*Oscillospira*, *Roseburia*) (Yang et al., 2021; Zhang et al., 2022) or are currently being used in this capacity (*Akkermansia*).^1^ Interestingly, the bacterial genera usually depleted in the presence of *Christensenellaceae* include several noxious taxa, for instance, opportunistic pathogens, such as *Klebsiella* and *Streptococcus*, which are known to cause infections in humans and animals, and *Fusobacterium* – a microorganism that can contribute to cancer (Rubinstein et al., 2019). Therefore, we hypothesize that *C. minuta* can modulate the gut microbiome by supporting the growth of beneficial species and inhibiting potentially harmful ones.

It is likely that different bacterial species interact through the transfer of metabolites. Ruaud et al. extensively studied the co-occurrence of the *Christensenellaceae* and the *Methanobacteriaceae* in the domain *Archaea*, the abundance of which was also inversely correlated with body mass index (Goodrich et al., 2014). They conducted a meta-analysis of 1,821 samples from 10 independent studies and confirmed a strong positive correlation at both the family level (between *Christensenellaceae* and *Methanobacteriaceae*) and the species level (between *C. minuta* and *Methanobrevibacter smithii*). Additionally, the authors co-cultured these two microorganisms to study their physical and metabolic interactions *in vitro*. They found that the strong co-occurrence patterns observed in the metagenomic studies were largely confirmed in the *in vitro* experiments. As an active H_2_ producer, *C. minuta* effectively supported the growth of *M. smithii*, which depends on H_2_ supply. In the co-culture, the amount of H_2_ released by *C. minuta*, was presumably sufficient to ensure the viability of *M. smithii*, equivalent to that in a monoculture with H_2_ excess. Moreover, *M. smithii* tended to form small inclusions within *C. minuta* flocs on solid media, as demonstrated by confocal microscopy, suggesting their mutually beneficial interaction. *M. smithii*, in turn, modulated the metabolism of *C. minuta*, causing a shift in SCFA production from butyrate to acetate. Based on the observed increase in acetate production, the authors conjectured that *C. minuta* could be an effective supplier of this substrate to other butyrate producers and directly facilitate their growth. They also hypothesized that methane production by *Methanobacteriaceae* results in carbon loss and less energy available to the host, which may in part explain the association between this bacterial family and the lean phenotype (Ruaud et al., 2020).

Members of the *Christensenellaceae* family are important components of an extremely complex, self-regulating community of intestinal microorganisms. Presumably, they interact in different ways. Some of them have been discovered, but most have not.

## 7 Commercial production of *C. minuta*-based new-generation probiotics

Probiotics are living organisms with specific requirements and often a high sensitivity to oxygen. Therefore, manufacturing next-generation probiotics on an industrial scale is an extremely demanding and time-consuming process, which differs drastically from a regular laboratory workflow. Cultured bacteria are not as resilient. For a probiotic drug to be effective, the bacteria must be kept alive by fine-tuning the environment (pH levels, temperature, atmospheric oxygen) and ensuring their viability and integrity throughout the process (drying, freezing, etc.). This applies in particular to anaerobic microorganisms.

Another challenge is ensuring that the drug is properly digested in the body. To reach their destination in the small and large intestine, beneficial bacteria must be resistant to low pH levels in the stomach. This potential challenge is usually addressed by overage, which compensates for losses during storage and drug passage through the gastrointestinal tract. Moreover, it is crucial to ensure freedom from allergens and/or potentially harmful substances. Therefore, any additional components (e.g., cryoprotectants) should be selected and handled with great care. Possible impurities from the culture media must be removed as far as possible. Quality control should be implemented at every stage of the production chain. Long-term stability is one of the most desirable properties of commercial drug products. An ideal probiotic drug is one that is moisture-free, has a good shelf life at ambient temperature for at least 12 months, and will produce significant numbers of viable microorganisms by the end of its shelf life. This can be achieved either through the use of protective techniques or by ensuring that the probiotic is highly resilient to stressful environmental conditions. Various techniques have been developed to ensure better survival of probiotic microorganisms, including spray drying, freeze drying, microencapsulation with alginates or other polysaccharides, etc. Microencapsulation has proven to be effective for a number of probiotic strains, such as *Akkermansia muciniphila* (77, 78), *Lactobacillus plantarum* (79), *Lactobacillus rhamnosus* (80, 81), *Bifidobacterium bifidum* (82) and others. Advances in delivery systems have enabled the incorporation of microorganisms into functional foods. For example, Marcial-Coba managed to incorporate microencapsulated *A. muciniphila* into dark chocolate, improving bacterial survival (83). Afzaal et al. incorporated microencapsulated *L. acidophilus* into ice cream and recovered viable bacteria after 120 days of storage (84). Several authors have also successfully used cheese to deliver various probiotic strains (85–87).

To the best of our knowledge, no attempt has been made to cultivate *C. minuta* on an industrial scale, and there have been no reports of the processing or stabilization of *C. minuta* as a probiotic drug or the development of an optimal drug formulation. *C. minuta* is a very promising probiotic agent, not only because of its therapeutic potential but also because it is relatively low-maintenance. It grows well on some fairly basic liquid and solid media and, most importantly, can tolerate oxygen for up to 24 h (2). This gives *C. minuta* a significant advantage over many other beneficial but strictly anaerobic microorganisms. Lower sensitivity to atmospheric oxygen provides more time for effective processing of raw *C. minuta* biomass, i.e., freeze-drying microencapsulation or incorporation into functional foods.

Another potentially promising form of *C. minuta*-based biotherapeutics is pasteurized *C. minuta* and its supernatant, which contains bacterial metabolites. To date, either live bacteria or their supernatants have been tested in all *in vivo* and *in vitro* experiments with *C. minuta* (2, 54, 55). Surprisingly, pasteurized *C. minuta* has remained unnoticed. Pasteurization is not a brand-new technique. It helps eliminate some challenges related to drug storage and delivery to the intestine. Pasteurized forms of another anaerobic strain, *Akkermansia muciniphila*, have been extensively tested and have demonstrated some exciting probiotic properties in both animal models (88) and humans (89). Therefore, pasteurized *C. minuta* and its supernatant are particularly attractive as candidates for biotherapeutics. Scaling up their production would likely be easier and cheaper than that of viable strains. Given the above benefits, further studies must include all three forms of *C. minuta* to identify an optimal form and improve our understanding of the mechanisms underlying its impressive effects.

## 8 Discussion

A new candidate NGP, *C. minuta*, has drawn the attention of the international scientific community since its isolation about 12 years ago. Many reports have suggested that there is a strong association between the species and the healthy and lean phenotype. However, this suggestion begs the question – which of the two is the cause and which is the effect? Do changes in the microbiome in general and in the abundance of *Christensenellaceae* in particular cause a disease or at least contribute to its development? Or does the disease, caused by reasons other than the microbiome, trigger microbial shifts? The causative role of *Christensenellaceae* in human diseases and its extent have yet to be established. One might think that the association between the depletion of this bacterial taxon and a disease is solely due to lifestyle and diet; hence, the microbial profile is an effect rather than the cause. There are a number of findings supporting this hypothesis. For instance, the abundance of *Christensenellaceae* increased in overweight patients after dietary changes (Aleman et al., 2018; Deledda et al., 2022; Jian et al., 2022) and increased consumption of animal products (David et al., 2014), but decreased in patients on a low-protein diet (Lai et al., 2019). Hence, the question of causality remains.

Nevertheless, some significant effects of *C. minuta* have been reported in more recent studies (Kropp et al., 2021; Mazier et al., 2021; Pan et al., 2022; Relizani et al., 2022), including the prevention of HFD-induced weight gain, restriction of gut inflammation, and preservation of gut permeability. Clearly, causality is a complex problem. Reducing it to a straightforward equation is of little value. Evidently, the microbiome and disease have a reciprocal relationship in which each affects the other. Hence, the emergence of any disorder is an intricate process involving numerous variables, and the microbiome is just one of them. Our commensal microbial communities are undoubtedly altered by pathological changes, and this can affect how a disease develops and progresses.

The initial *in vivo* and *in vitro* experiments have demonstrated the exceptional value of *C. minuta* as a candidate NGP. Because of its relative tolerance to atmospheric oxygen and, consequently, much easier handling, *C. minuta* has an advantage over other anaerobic strains.

The formulation and manufacturing of a commercial NGP derived from *C. minuta* present a number of scientific and legal challenges. First and foremost, further research should corroborate the current results and should explore new hypotheses. We believe many diseases could also be treated with *C. minuta*-derived NGP; however, metabolic disorders and inflammatory bowel disease should probably still be its primary targets. *C. minuta* may prove to be of great benefit to patients with atopic disorders because it can improve gut permeability and mitigate systemic inflammation. Patients with asthma (Benard et al., 1996), eczema (Jackson et al., 1981; Pike et al., 1986), and food allergies (Schrander et al., 1990) are more likely to have a “leaky gut,” which is thought to contribute to disease pathogenesis and trigger flares. Given its ability to limit intestinal stem cell proliferation (Kaiko et al., 2016), a *C. minuta*-derived probiotic may also be beneficial for patients with malignancies, especially colon cancer. Hence, we believe *C. minuta* should be used to treat a variety of conditions, which calls for more research using animal models.

Second, *C. minuta* in forms other than live strains also deserves more consideration. Compared to viable *C. minuta*, the *C. minuta* metabolites (supernatant) have demonstrated impressive effects both in *in vitro* and *in vivo* experiments (Kropp et al., 2021). Surprisingly, no reports on experiments using pasteurized *C. minuta* exist, suggesting a gap in this field of study. In our opinion, both pasteurized *C. minuta* and its postbiotic (supernatant) merit further consideration, along with viable *C. minuta*. Indeed, testing several therapeutic forms of *C. minuta* rather than one live form would greatly facilitate unraveling its mechanisms of action. For example, a more detailed analysis of the *C. minuta* metabolites using high-performance liquid chromatography or other techniques could shed some light on how this microorganism has become so important for human health and would expedite the development of a formulation. Given the challenges associated with the large-scale production of NGPs, especially live biotherapeutics, selecting the most optimal and cost-effective form is crucial.

Third, it is critical to determine whether any form of *C. minuta* can produce long-term effects on the gut microbiome and on the entire body. So far, very few studies have attempted to analyze the effects of *C. minuta*-based treatment. Mazier et al. examined the effects of *C. minuta* in a SHIME^®^ model and found no *C. minuta* engraftment into the microbial community of the simulated intestine, although some effects lasted during a one-week wash-out period (Mazier et al., 2021). We believe that more studies on animals with a longer wash-out period are needed to gain a better understanding of the long-term effects of a *C. minuta*-based pro/postbiotic.

Finally, we would like to emphasize the potential utility of mixed biotherapeutics containing multiple microbial species. The *Christensenellaceae* family has demonstrated strong correlations with other potentially beneficial strains; therefore, we hypothesize that combining it with other NGPs, such as *Akkermansia muciniphila* and *Roseburia faecis*, or even with conventional probiotic strains, such as bifidobacteria and lactobacilli, may enhance the treatment effects. In fact, people with disorders often experience depletion of many bacterial taxa, which could be remedied by a combination of probiotics. These could be mixtures of at least one viable probiotic strain and at least one postbiotic from a different strain (or strains). To our knowledge, only mixtures of conventional probiotic strains have been tested (Chapman et al., 2006) or formulated so far, whereas combinations of NGPs remain largely uninvestigated. We surmise that this approach will advance biotherapeutics and pave the way for new multicomponent and multi-purpose pro/postbiotics, which are highly likely to include *C. minuta* as one of the most important representatives of a healthy gut microbiome.

Once the best or most cost-effective form/combination has been identified, other, more practical challenges should be addressed, such as scaling up the production, safety, and clinical trials. Clearly, only randomized placebo-controlled trials will suffice to demonstrate the efficacy of any *C. minuta*-containing biotherapeutic. Since biotherapeutic drugs are based on living microorganisms, there will be additional regulatory challenges, and the development and registration processes will be complicated. To date, only one pharmaceutical company, YSOPIA Bioscience (France), has attempted to reach these final stages with their single-strain live Xla1 biotherapeutic containing *C. minuta* DSM 33407. They reported entering the first in-human clinical trials in 2021 and shared the legal challenges they had met in advancing to the clinical trials stage (Paquet et al., 2021).

Thus, significant progress has been made in developing effective and reliable NGPs, and a *C. minuta*-derived biotherapeutic seems to be within reach. However, the promising findings of numerous studies must be confirmed in clinical trials.

## Author contributions

OI, DT, AKo, and LM: literature search, manuscript preparation, writing, and editing. MT: figures and manuscript editing. DK, AZ, MI, VY, VM, AKe, SK, and SY: manuscript discussion and editing. VY, VM, AKe, SK, and SY: overall supervision. All authors contributed to the article and approved the submitted version.

## Footnotes

^1^https://pendulumlife.com/products/pendulum-akkermansia?dsc_appld=true
